# Clinical Significance of Fragile X Syndrome 2 (FXR2) in Breast Cancer

**DOI:** 10.3390/genes16030302

**Published:** 2025-03-01

**Authors:** Ohud A. Alsalmi, Abrar I. Aljohani, Shahad M. Almutairi, Rana O. Alsufyani, Abdulrahman R. Alrubayee, Khalid J. Alzahrani, Ghaida E. Alkhammash, Hessa M. Aljuaid, Hanan S. Alghamdi, Fouzeyyah A. Alsaeedi

**Affiliations:** 1 Department of Clinical Laboratory Sciences, College of Applied Medical Sciences, Taif University, Taif 21944, Saudi Arabia; abrar.g@tu.edu.sa (A.I.A.); s44101045@students.tu.edu.sa (S.M.A.); s44100389@students.tu.edu.sa (R.O.A.); ak.jamaan@tu.edu.sa (K.J.A.); s44480487@students.tu.edu.sa (G.E.A.); s44100039@students.tu.edu.sa (H.M.A.); s44580159@students.tu.edu.sa (H.S.A.);; 2 Medical Oncology Department, King Abdulaziz Specialist Hospital, Taif 26521, Saudi Arabia; aalrubayee@moh.gov.sa

**Keywords:** FXR2, breast cancer, clinical significance

## Abstract

**Background**: The fragile X protein family comprises three members: the fragile X syndrome protein (FMRP) and its structural homologs, fragile X syndrome 1 and 2 (FXR1 and FXR2). FMRP has a significant role in controlling the genesis and progression of various forms of human cancer. However, studies on the prognostic significance of FXR2 in cancer are scarce. Thus, this study aimed to investigate the clinicopathological significance of FXR2, a member of the FMRP family, in primary breast cancer (BC). **Methods**: A total of 100 formalin-fixed paraffin-embedded (FFPE) tissue blocks from invasive BC cases were collected from King Abdulaziz Hospital in Saudi Arabia. Immunohistochemistry (IHC) was used to assess FXR2 protein expression in the BC tissues, and the results were correlated with clinicopathological parameters, such as tumor grade, tumor size and hormone receptor status. Additionally, the association between clinicopathological features and *FXR2* mRNA expression was assessed using the BC Gene-Expression Miner v5.0 tool on all publicly available DNA microarray (*n* = 10,872) and RNA sequence (*n* = 4421) data to validate the results. **Results**: FXR2 protein expression was significantly associated with human epidermal growth factor 2 (HER2) negativity (*p* = 0.010) and low Ki67 (*p* < 0.001). Both DNA microarray and RNA sequence data showed that HER2 negativity was strongly linked to high levels of *FXR2* mRNA. High *FXR2* mRNA levels were also correlated with hormone receptor negativity and mutated p53. **Conclusions**: This study suggests that FXR2 may have indirect clinical significance in BC. However, further studies are warranted to deepen our understanding of the association between FXR2 and other clinicopathological parameters, which could lead to improved diagnostic, treatment, and prognostic strategies for BC patients.

## 1. Introduction

Breast cancer (BC) is a major public health issue, affecting millions of women annually and constituting one of the most prevalent cancers worldwide [1]. It is characterized by a high degree of heterogeneity, exhibiting a wide range of tumor behaviors and therapeutic responses due to variations in gene mutations, gene expression, and epigenetic modifications [2]. The hormone receptor status of BC is both a prognostic and predictive factor. Tumors that express estrogen receptor (ER) and/or progesterone receptor (PR) have an excellent prognosis for early-stage disease and reduced death rates for advanced disease [3]. Tumors that overexpress the receptor tyrosine kinase human epidermal growth factor 2 (HER2) have a worse outcome [4]. Targeted endocrine therapy or anti-HER2 therapy can be used to treat people whose tumors express or overexpress any of these three receptors [3]. However, not all Her2 cancers respond to anti-Her2 therapies. On the other hand, tumors that have a negative expression of these hormone receptors have worse clinical outcomes. This is especially true for triple-negative breast cancer (TNBC), which has fewer treatment options than other tumors [5]. In clinical settings, prognostic or predictive factors can guide the selection of systemic therapy and predict outcomes [6]. Thus, the identification of biomarkers is gaining significance in medical research, particularly since scientific advancements have enhanced our understanding of diseases and genetics, resulting in more tailored therapies [7].

The fragile X-related (FXR) gene family is composed of three genes that encode highly homologous RNA binding proteins (RBPs): fragile X syndrome 1 (FMR1), FMR1 autosomal homolog 1 (FXR1), and FMR1 autosomal homolog 2 (FXR2). These genes are located on chromosomes Xq27.3, 3q26.33, and 17p13.1, respectively [8]. The FXR family members exhibit analogous structures, particularly in their N-terminal and core domains, which are primarily responsible for protein–protein interactions and activities [9]. Nevertheless, the C-termini of these proteins exhibit considerable variation [8]. FXR1, FXR2, and FMR1 can interact with one another. Despite their ability to transit between the cytoplasm and nucleus, their primary location within cells is the cytoplasm, where they bind with ribosomes to form ribonucleoprotein (RNP) complexes [10]. The FXR gene family has a crucial role in binding and regulating mRNA stability, transportation, and translation [11].

Converging data from a few studies underscores the involvement (either direct or indirect) of fragile X syndrome protein (FMRP) in cancer. FXR1 has emerged as an oncogenic protein, or at least a facilitator of oncogenesis that promotes carcinomas in diverse tissues, more so than the other two proteins. In addition, FXR1 overexpression is a significant indicator of unfavorable outcomes in many malignancies [8]. In comparison to FXR1 and FMRP, the role of FXR2 in cancer remains largely unexplored. According to the Human Protein Atlas, the expression of FXR2 shows limited specificity in cancer; nonetheless, FXR2 overexpression appears advantageous for the survival of patients with pancreatic cancer [12].

There are gaps in our understanding of the FXRP family, particularly FXR2 and its involvement in cancer, necessitating future research to promote the translation of insights from bench to bedside. The understanding of FXR2’s clinical significance is still at a very preliminary stage. Thus, the primary aim of this study was to investigate the clinical implications of FXR2 in primary BC, with the goal of enhancing prognostic accuracy, facilitating more effective disease monitoring, and enabling the development of more precisely tailored therapeutic strategies.

## 2. Materials and Methods

### 2.1. FXR2 Protein Analysis Study Cohort

This study evaluated a cohort of 100 BC patients from the Histopathology Department of King Abdul Aziz Specialist Hospital (KASH) in Saudi Arabia. Formalin-fixed paraffin-embedded (FFPE) tissue blocks were retrieved for those patients. The accessed clinicopathological features, including patients’ age, tumor grade, tumor size, tumor stage, Ki-67, hormone receptor status, and molecular subtypes, were analyzed. The status of hormone receptors, including ER, PR, and HER2, was assessed using immunohistochemistry (IHC), categorizing tumors as ER+/PR+ when staining intensity exceeded 1%. The tumor was classified as HER2-positive if it exhibited a score of 3+ via IHC or 2+ via fluorescence in situ hybridization, signifying *HER2* gene amplification [13]. Ki67 positivity was considered when more than 20% of the tumor cells were positive. By using St. Gallen’s surrogate classification for BC based on the IHC profile, this study identified four types of BC, including luminal A, luminal B, HER2-positive, and TNBC [14], as follows:

- **Luminal A**: ER-positive and/or PR-positive, HER2-negative, low proliferation (Ki67% < 20%).
- **Luminal B**: ER-positive and/or PR-positive, HER-2-negative or -positive, high proliferation (Ki67% ≥  20%).
- **HER2**: ER-negative and/or PR-negative, HER2-positive.
- **TNBC**: ER-negative and/or PR-negative, HER2-negative.

A correlation between these parameters and FXR2 protein expression was determined. This study was conducted with the approval of the Research and Studies Department of KASH (approval number HAP-02-T-067). All participants provided informed consent. This research was performed in compliance with the Declaration of Helsinki.

### 2.2. A Comprehensive Transcriptomics Analysis of FXR2 in BC

BC Gene-Expression Miner v5.0, a statistical mining tool for published annotated BC transcriptome data, comprising DNA microarrays (*n* = 10,872) and RNA sequences (*n* = 4421), was used to assess the expression of *FXR2* mRNA [15]. An assessment of *FXR2* mRNA expression and its correlation with a variety of aggressive features of BC, such as molecular subtype, tumor grade, and tumor size, was performed.

### 2.3. Validation of FXR2 Transcriptomics Analysis: Immunohistochemical Staining for FXR2 in FFPE Tissue Sections

**Figure 1:**
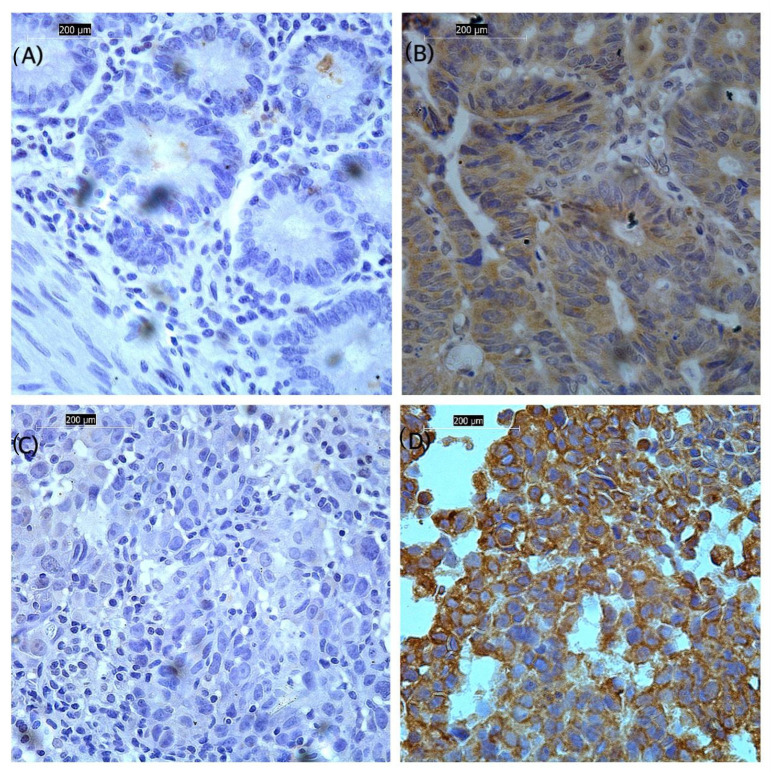
Light microscope images (magnification ×40) show the immunohistochemical protein expression of FXR2 in breast tissue. FXR2 expression was observed in the cytoplasm. (**A**) Negative control of colon tissue without FXR2 antibody. (**B**) Colon cancer sample used as a positive control. (**C**) Invasive breast carcinoma showing low expression. (**D**) Invasive breast carcinoma showing high expression. High expression is defined by an H-score cutoff point of 110 or more, based on the median for dichotomization.

The FFPE samples were stained with an optimized FXR2 antibody using an IHC protocol. The tissue sections were prepared, dewaxed, rehydrated, and treated with hydrogen peroxide (H_2_O_2_, Fisher Scientific, H/1750, Loughborough, The United Kingdom (UK)) to block endogenous peroxidase. The manufacturer’s instructions were followed for antibody retrieval, and antigen retrieval was performed using citrate buffer at pH 6. Sections were blocked in a blocking solution containing 2% (*w*/*v*) bovine serum albumin (Sigma-A4042, St. Louis, MO, USA) to prevent non-specific binding and then incubated with a 1:10 diluted FXR2 rabbit polyclonal primary antibody (Sigma, HPA018246, St. Louis, MO, USA) at room temperature for 1 h. After washing, sections were incubated in a 1:200 dilution of a biotinylated anti-mouse secondary antibody (Vector Laboratories, PK-6102, Newark, NJ, USA) and avidin–biotin complex (ABC, Vector Labs, PK-6100, Newark, NJ, USA). Diaminobenzidine was subsequently applied to the sections (Vector Laboratories, SK-4100, Newark, NJ, USA). Slides were counterstained with Mayer’s hematoxylin (Sigma, MHS16, St. Louis, MO, USA), dehydrated, and mounted. Negative and positive controls were incorporated in IHC. As a negative control, the primary antibody was omitted from the tissue. A tissue section from colon cancer was stained as a positive control, as recommended by the antibody manufacturer (Figure 1A,B).

### 2.4. Scoring

Stained sections were evaluated utilizing light microscopy (Leica Microsystems, Leica DMI 3000B, Watzlar, Germany) at a magnification of 40×. The semi-quantitative evaluation of FXR2 cytoplasmic expression was performed with the modified histochemical score (H-score), which integrated staining intensity (ranging from 0 to 3+) and the percentage of stained cells (ranging from 0 to 1). This resulted in a final score ranging from 0 to 300 [13]. To ensure accuracy, the scoring process was independently performed by two individuals, minimizing potential biases or errors and enhancing the reliability of the results. For FXR2 and immunoscoring, a substantial concordance among the evaluators was noted (interclass correlation coefficient [ICC] = 0.90, *p* < 0.001). Tumors were categorized as FXR2-low or FXR2-high based on the median score (H-score of 110) as the data did not follow a normal distribution, which served as a predefined cutoff for classification.

### 2.5. Statistical Analysis

Statistical analysis was conducted using SPSS Version 24.0 (SPSS, Chicago, IL, USA). The ICC test was used to determine the degree of concordance between the two observers’ FXR2 scores. To assess the correlation between FXR2 expression and clinicopathological parameters, a univariate analysis employing the chi-square test was performed. Statistical significance was established at a *p*-value < 0.05.

## 3. Results

### 3.1. Expression of FXR2 mRNA

**Figure 2:**
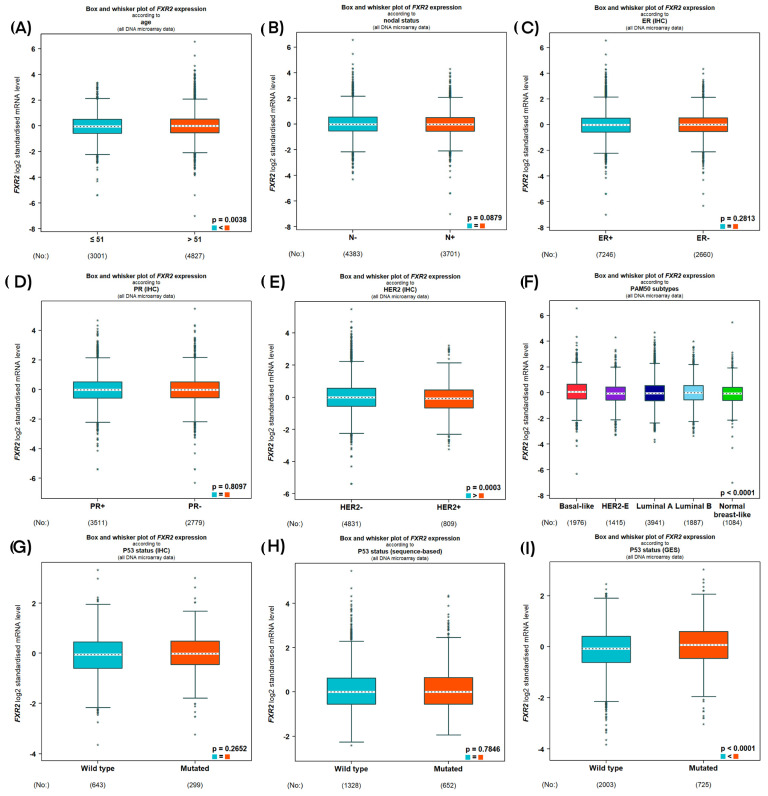
*FXR2* mRNA expression in relation to breast cancer clinical features based on DNA microarray data. Correlation of *FXR2* mRNA with (**A**) age, (**B**) nodal status, (**C**) ER (IHC), (**D**) PR (IHC), (**E**) HER2 (IHC), (**F**) PAM50 molecular subtypes (more significant in basal-like and luminal A subgroup), (**G**) P53 gene status (IHC), (**H**) P53 gene status (sequence-based) and (**I**) P53 gene status (GES).

Initially, we used BC Gene-Expression Miner v5.0 to evaluate the association between *FXR2* and aggressive features of BC at the mRNA level. DNA microarray data showed that HER2 negativity was strongly linked to high levels of *FXR2* mRNA (*p* = 0.0003; Figure 2E), which is consistent with the FXR2 protein expression results. Interestingly, the expression level of *FXR2* mRNA was noted to be higher in the *P53*-mutated group when *P53* was evaluated based on gene expression signature (GES; *p* < 0.0001; Figure 2I). However, when *P53* was evaluated based on IHC or sequences, no significant correlation was observed between the expression of *FXR2* mRNA and *P53* status (Figure 2G,H).

**Figure 3:**
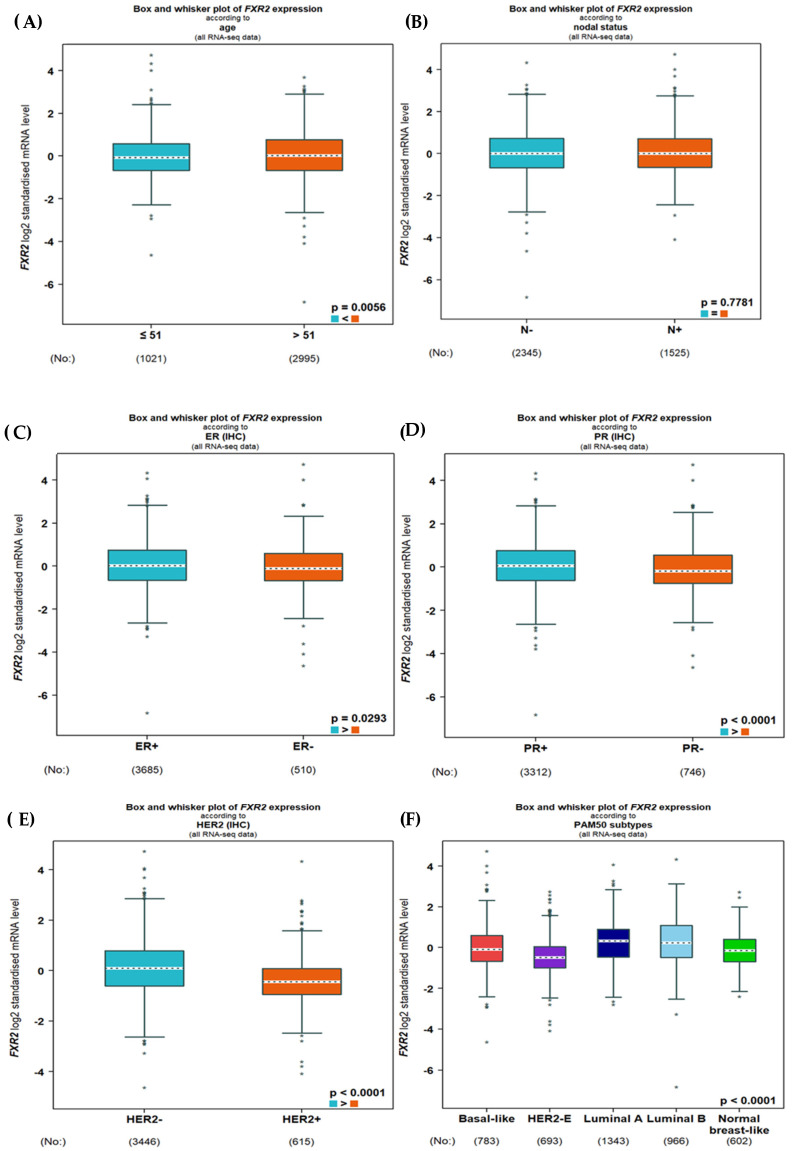
*FXR2* mRNA expression in relation to breast cancer clinical features based on RNA sequence data. Correlation of *FXR2* mRNA with (**A**) age, (**B**) nodal status, (**C**) ER (IHC), (**D**) PR (IHC), (**E**) HER2 (IHC), and (**F**) PAM50 molecular subtypes (more significant in luminal B and basal-like subgroups).

Regarding RNA sequence data, a high *FXR2* mRNA level was associated with hormone receptor negativity (ER: *p* = 0.0293; PR: *p*= 0.0001; and HER2-negative: *p* = 0.009; Figure 3C–E).

Regarding IHC subtypes, the high *FXR2* mRNA level in both sets of transcriptomic data was mostly correlated with TNBC and the luminal subtypes, followed by the other subtypes (*p* < 0.0001; Figure 2 and Figure 3F). No significant correlations were observed with the other clinicopathological parameters.

### 3.2. Association of FXR2 Protein Expression Level with Clinicopathological Parameters

To validate the results of the transcriptomics analysis of *FXR2* in BC, FXR2 protein expression was examined on BC samples using the IHC technique. The expression of FXR2 was found in the cytoplasm of invasive breast tumors, with no obvious membranous or nuclear staining and intensity ranging from nonexistent to high (Figure 1C,D). The high expression of the FXR2 protein was significantly correlated with HER2 negativity (*p* = 0.008) and low Ki67 (*p* < 0.001) (Table 1). Interestingly, among the IHC molecular subtypes, a high level of FXR2 was associated with the triple-negative, HER2+, and luminal A subtypes, followed by the luminal B subtype (*p* < 0.001; Table 1). Although no other significant correlations were found with the remaining clinicopathological parameters, FXR2 can be seen to be overexpressed in tumor sizes ≥ 10 mm (64.3%), in TNM stage IIB (90.9%), and in negative lymph nodal status (64%) (Table 1).

## 4. Discussion

BC is well known as a complex and heterogeneous condition, with the prognosis differing by subgroup [16]. This complexity hinders a comprehensive understanding of BC biology and, consequently, the development of a therapeutic strategy for the disease [17]. Identifying biomarkers associated with the onset of early-stage BC could facilitate the assessment of metastatic risk and inform treatment strategies.

The FXR family proteins FMRP, FXR1, and FXR2 are RBPs essential for RNA metabolism. Recent analytical discoveries indicate divergent roles of this protein family in fragile X syndrome (FXS) and carcinogenesis, contingent upon their expression patterns in human tissues [8]. FXR1 can promote tumorigenesis and prevent senescence in certain types of cancer, such as head and neck squamous cell carcinoma and lung cancer [18]. Additionally, the overexpression of FXR1 is a strong indicator of a poor outcome in several types of cancer [19]. However, little is known regarding the function of FXR2 in cancer compared to FXR1 and FMRP. Knowledge of the clinical relevance of FXR2 in cancer, particularly BC, is primitive. To date, no study has investigated the significance of FXR2 in cancer progression. Thus, this study’s principal objective was to examine the clinical significance of FXR2 in primary BC, aiming to improve prognostic precision, enhance disease monitoring, and foster the development of more specifically tailored therapeutic approaches.

This study revealed that FXR2 protein expression was significantly associated with HER2 negativity. In concordance with FXR2 protein expression, transcriptomic analyses of DNA microarray and RNA sequence data revealed the same association between high *FXR2* mRNA expression and HER2-negative BC. HER2-negative cancer cells may exhibit slower growth and a reduced likelihood of recurrence or metastasis compared to cancer cells with elevated surface HER2 expression [20]. Several recent studies suggest that HER2-low and HER-negative BC may be different disease entities. A better understanding of this new subtype may allow a large number of patients to benefit from HER2-targeted therapy. However, data on the clinical distinction between the groups are currently lacking, along with an incomplete comprehension of the biology of HER2-negative BC [21]. Thus, the association between the high expression of FXR2 and HER2-negative BC may suggest that FXR2 is linked to a new BC subtype or may not be linked to aggressive features of BC.

**Figure 4:**
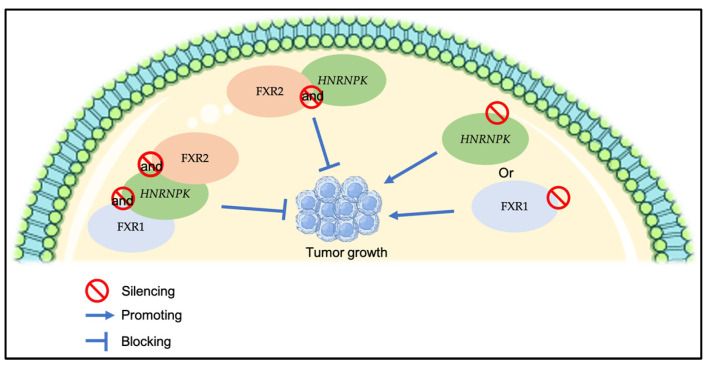
A summary of the effect of silencing the RNP complex composed of RBPs on tumor growth.

High FXR2 protein expression was also correlated with low Ki67. Ki67 expression is recognized as a marker of oncogenesis, closely linked to aggressive tumor traits, proliferation, and reduced survival rates [22]. Notably, although no other statistically significant correlations were identified with the other clinicopathological criteria, FXR2 is observed to be overexpressed in tumors measuring ≥ 10 mm, in TNM stage IIB, and in negative lymph nodal status. This suggests that FXR2 may initially increase to inhibit tumor growth, but it could subsequently lose its suppressive role in later stages, potentially due to its co-expression with other genes. This is supported by a previous study demonstrating that the silencing of a new RNP complex composed of RBPs (*HNRNPK*) or *FXR1* augmented primary tumor growth, while the simultaneous silencing of *HNRNPK* and *FXR2* or the triple silencing of *HNRNPK*, *FXR1*, and *FXR2* markedly reduced primary tumor growth, suggesting that the co-expression of *HNRNPK* and *FXR2* is essential for in vivo primary tumor growth (Figure 4) [23]. The functional role of FXR2 at the early and late stages of BC necessitates more investigation.

Furthermore, in the DNA microarray data, the expression level of *FXR2* mRNA was observed to be elevated in the *P53* mutant when *P53* was evaluated based on GES. However, this was not the case when *P53* was evaluated based on sequences or IHC. The difference in the results of the association between *P53* and *FXR2* on the GES, sequence and IHC level may be explained by a previous study that demonstrated that *P53* mutants may be classified as wild-type-like, which are *P53* mutant variants with a reduced biological impact at the sequence level [24]. *P53* mutations have a variety of effects on cancer. Some mutations result in a complete loss of function, while others exert a dominant-negative effect (such as the transdominant suppression of wild-type *p53* or oncogenic gain of function). Additionally, certain mutations result in a partial loss of function, in which only a fraction of the *p53* target genes are dysregulated [24,25]. An interesting relationship between *TP53* and *FXR2* has been reported in the literature [26]. Investigating databases of DNA sequences from human cancer cells from liver hepatocellular carcinoma, stomach adenocarcinoma, and lung adenocarcinoma showed that the loss of the *TP53* gene is often linked to the loss of the gene *FXR2* [26]. This result is attractive because the suppression of the remaining family member FXR1 preferentially inhibits cell growth in human cancer cells with homozygous deletions of both *TP53* and *FXR2* through a collateral lethality mechanism [21]. Overall, the association between the high level of *FXR2* and the *P53* mutant may exhibit different effects in BC. However, a further exploration is warranted to elucidate the clinical importance of FXR2 regarding *P53* status.

Although no association between FXR2 and aggressive tumor features was present in this study, high FXR2 expression was associated with the TNBC molecular subtype. TNBC is an aggressive subtype distinguished by its extensive intra-tumoral heterogeneity and tendency to develop resistance to therapies [27]. Several genetic alterations can be involved in TNBC [27]. This could explain the correlation between FXR2 and TNBC, which could be attributed to the heterogeneity of the tumor cells and the co-expression of certain genes with FXR2. In addition, the high expression of FXR2 does not imply that FXR2 is oncogenic; rather, FXR2 may be a tumor suppressor that is associated with elevated *TP53* mutations. Mutations or deletions of both genes may be required to boost oncogenic efficiency.

Although FXR2 expression has little specificity in cancer, the Human Protein Atlas indicates that the overexpression of FXR2 is a good prognostic marker in pancreatic cancer [12]. The translational regulatory RNA (treRNA) of long non-coding RNAs was demonstrated to be elevated in matched samples of original BC and lymph node metastases, and its expression promoted tumor invasion. treRNA produces a new RNP complex with RBPs (hnRNP K, FXR1, and FXR2), PUF60, and SF3B3, which is essential for RNA to function [23]. This suggests that FXR2 may depend on other genes in cancer progression.

Despite significant progress in understanding FXS, knowledge of the modulation of FXR protein family members, especially FXR2, and their regulation of key genes in cancer remains nascent. This study demonstrates that FXR2 may have indirect clinical significance in BC. FXR2 may function as a tumor suppressor linked to increased *TP53* mutations. FXR2 may also enhance oncogenic efficiency when deletions of certain genes, such as *P53*, occur. Further functional investigations are required to further increase our understanding of the role of FXR2 in BC.

Notwithstanding the significant findings of this investigation, the main limitation of this study is that it was based on a small clinical sample size. Another limitation of this study is the small number of FXR2 studies in the cancer field. Despite these limitations, this work was the first to examine the significance of FXR2 in BC and offers valuable insights into its clinical significance in BC. This would be a fruitful area for further work, including in vivo and functional studies, to increase our understanding of the role of FXR2 in BC, which could lead to better diagnostic, therapeutic, and prognostic strategies for BC patients.

## Figures and Tables

**Table 1:** Statistical correlations between FXR2 protein expression and clinicopathological factors in the King Abdul-Aziz Specialist Hospital breast cancer cohort (*n* = 100).

Clinicopathological Parameters	FXR2 Protein Expression		*p*-Value
	Low (*n* = 44) n (%)	High (*n* = 56) n (%)	
**Age (years)**			
<50	25 (53.2)	22 (46.8)	0.548
≥50	25 (47.2)	28 (52.8)	
**Tumor size**			
<10 mm	17 (54.8)	14 (45.2)	0.141
≥10 mm	10 (35.7)	18 (64.3)	
**Tumor grade**			
I	3 (37.5)	5 (62.5)	0.271
II	26 (46.4)	30 (53.6)	
III	19 (63.3)	11 (36.7)	
**TNM stages**			
Stage I	4 (44.4)	5 (55.6)	0.117
Stage IIA	10 (50)	10 (50)	
Stage IIB	1 (9.1)	10 (90.9)	
Stage IIIA	4 (80)	1 (20)	
Stage IIIB	2 (50)	2 (50)	
Stage IIIC	0 (0)	1 (100)	
Stage IV	6 (60)	4 (40)	
**Lymph nodal status**			
Negative	9 (36)	16 (64)	0.200
Positive	14 (53.8)	12 (46.2)	
**Estrogen receptor (ER), IHC**			
Negative	2 (9.1)	20 (90.9)	0.369
Positive	13(16.9)	64 (83.1)	
**Progesterone receptor (PR), IHC**			
Negative	3 (12)	22 (88)	0.611
Positive	12 (16.2)	62 (83.8)	
**Human Epidermal Growth Factor Receptor 2 (HER2), IHC**			
Negative	30 (41.1)	43 (58.9)	0.008
Positive	18 (72)	7 (28)	
**Ki67 (IHC)**			
Negative (<20)	10 (26.3)	28 (73.7)	<0.001
Positive (>20)	37 (64.9)	20 (35.1)	
**Immunohistochemistry subtype**			
Luminal A	10 (25.6)	29 (74.4)	<0.001
Luminal B	34 (94.4)	2 (5.6)	
HER2-positive	1 (12.5)	7 (87.5)	
Triple negative	2 (14.3)	12 (85.7)	

## Data Availability

The datasets presented in this article are not readily available because privacy or ethical restrictions. Data cannot be provided without the permission of the Directorate of Health Affairs.

## References

[1] Łukasiewicz S., Czeczelewski M., Forma A., Baj J., Sitarz R., Stanisławek A. (2021). Breast Cancer-Epidemiology, Risk Factors, Classification, Prognostic Markers, and Current Treatment Strategies—An Updated Review. Cancers.

[2] Lüönd F., Tiede S., Christofori G. (2021). Breast cancer as an example of tumour heterogeneity and tumour cell plasticity during malignant progression. Br. J. Cancer.

[3] McAndrew N., Finn R. (2022). Clinical Review on the Management of Hormone Receptor-Positive Metastatic Breast Cancer. JCO Oncol. Pract..

[4] Carey L.A., Perou C.M., Livasy C.A., Dressler L.G., Cowan D., Conway K., Karaca G., Troester M.A., Tse C.K., Edmiston S. (2006). Race, breast cancer subtypes, and survival in the Carolina Breast Cancer Study. JAMA.

[5] Nielsen T.O., Hsu F.D., Jensen K., Cheang M., Karaca G., Hu Z., Hernandez-Boussard T., Livasy C., Cowan D., Dressler L. (2004). Immunohistochemical and clinical characterization of the basal-like subtype of invasive breast carcinoma. Clin. Cancer Res..

[6] Krystel-Whittemore M., Tan P.H., Wen H.Y. (2024). Predictive and prognostic biomarkers in breast tumours. Pathology.

[7] Simms L., Barraclough H., Govindan R. (2013). Biostatistics primer: What a clinician ought to know--prognostic and predictive factors. J. Thorac. Oncol..

[8] Majumder M., Johnson R.H., Palanisamy V. (2020). Fragile X-related protein family: A double-edged sword in neurodevelopmental disorders and cancer. Crit. Rev. Biochem. Mol. Biol..

[9] Ramos A., Hollingworth D., Adinolfi S., Castets M., Kelly G., Frenkiel T.A., Bardoni B., Pastore A. (2006). The structure of the N-terminal domain of the fragile X mental retardation protein: A platform for protein-protein interaction. Structure.

[10] Hoogeveen A.T., Willemsen R., Oostra B.A. (2002). Fragile X syndrome, the Fragile X related proteins, and animal models. Microsc. Res. Tech..

[11] Chen E., Sharma M.R., Shi X., Agrawal R.K., Joseph S. (2014). Fragile X mental retardation protein regulates translation by binding directly to the ribosome. Mol. Cell.

[12] Uhlén M., Zhang C., Lee S., Sjöstedt E., Fagerberg L., Bidkhori G., Benfeitas R., Arif M., Liu Z., Edfors F. (2017). A pathology atlas of the human cancer transcriptome. Science.

[13] Cizkova K., Foltynkova T., Gachechiladze M., Tauber Z. (2021). Comparative Analysis of Immunohistochemical Staining Intensity Determined by Light Microscopy, ImageJ and QuPath in Placental Hofbauer Cells. Acta Histochem. Cytochem..

[14] Kunheri B., Raj R.V., Vijaykumar D., Pavithran K. (2020). Impact of St. Gallen surrogate classification for intrinsic breast cancer sub-types on disease features, recurrence, and survival in South Indian patients. Indian J. Cancer.

[15] Jézéquel P., Gouraud W., Ben Azzouz F., Guérin-Charbonnel C., Juin P.P., Lasla H., Campone M. (2021). bc-GenExMiner 4.5: New mining module computes breast cancer differential gene expression analyses. Database.

[16] Alanyalı S.D., Haydaroglu A., Ozyigit G. (2013). Prognostic and Predictive Factors. Principles and Practice of Modern Radiotherapy Techniques in Breast Cancer.

[17] Fumagalli C., Barberis M. (2021). Breast Cancer Heterogeneity. Diagnostics.

[18] Majumder M., House R., Palanisamy N., Qie S., Day T.A., Neskey D., Diehl J.A., Palanisamy V. (2016). RNA-Binding Protein FXR1 Regulates p21 and TERC RNA to Bypass p53-Mediated Cellular Senescence in OSCC. PLoS Genet..

[19] Cao H., Gao R., Yu C., Chen L., Feng Y. (2019). The RNA-binding protein FXR1 modulates prostate cancer progression by regulating FBXO4. Funct. Integr. Genom..

[20] Harbeck N., Gnant M. (2017). Breast cancer. Lancet.

[21] Gamrani S., Akhouayri L., Boukansa S., Karkouri M., El Fatemi H. (2023). The Clinicopathological Features and Prognostic Significance of HER2-Low in Early Breast Tumors Patients Prognostic Comparison of HER-Low and HER2-Negative Breast Cancer Stratified by Hormone Receptor Status. Breast J..

[22] Davey M.G., Hynes S.O., Kerin M.J., Miller N., Lowery A.J. (2021). Ki-67 as a Prognostic Biomarker in Invasive Breast Cancer. Cancers.

[23] Gumireddy K., Li A., Yan J., Setoyama T., Johannes G.J., A Ørom U., Tchou J., Liu Q., Zhang L., Speicher D.W. (2013). Identification of a long non-coding RNA-associated RNP complex regulating metastasis at the translational step. EMBO J..

[24] Miller L.D., Smeds J., George J., Vega V.B., Vergara L., Ploner A., Pawitan Y., Hall P., Klaar S., Liu E.T. (2005). An expression signature for p53 status in human breast cancer predicts mutation status, transcriptional effects, and patient survival. Proc. Natl. Acad. Sci. USA.

[25] Monti P., Campomenosi P., Ciribilli Y., Iannone R., Inga A., Abbondandolo A., Resnick M.A., Fronza G. (2002). Tumour p53 mutations exhibit promoter selective dominance over wild type p53. Oncogene.

[26] Fan Y., Yue J., Xiao M., Han-Zhang H., Wang Y.V., Ma C., Deng Z., Li Y., Yu Y., Wang X. (2017). FXR1 regulates transcription and is required for growth of human cancer cells with *TP53/FXR2* homozygous deletion. Elife.

[27] So J.Y., Ohm J., Lipkowitz S., Yang L. (2022). Triple negative breast cancer (TNBC): Non-genetic tumor heterogeneity and immune microenvironment: Emerging treatment options. Pharmacol. Ther..

